# Supplementary material for: High quality factor metasurfaces for two-dimensional wavefront manipulation
Source: Nat Commun. 2023 Dec 20;14:8476. doi: 10.1038/s41467-023-44164-4 (PMC10733294; doi:10.1038/s41467-023-44164-4)
Supplement: Supplementary file 1 — Supplementary Info [file 41467_2023_44164_MOESM1_ESM.pdf]

Supplementary Information:

High quality factor metasurfaces for two-dimensional wavefront manipulation

Authors

Claudio U. Hail[1], Morgan Foley[2], Ruzan Sokhoyan[1], Lior Michaeli[1], Harry A. Atwater[1]*

Affiliations

[1] Thomas J. Watson Laboratory of Applied Physics, California Institute of Technology, Pasadena, California 91125

[2] Department of Physics, California Institute of Technology, Pasadena, California 911125

Supplementary Figures

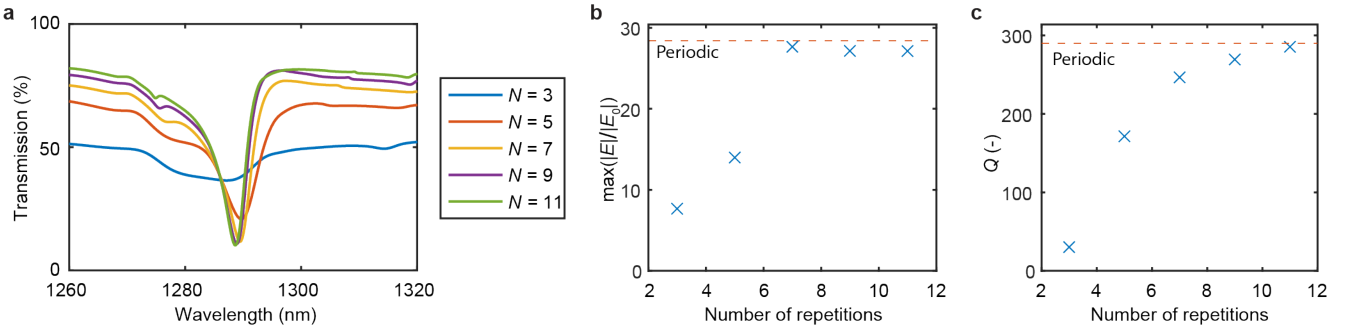

Supplementary Figure 1 | Array-size dependence. a, Simulated transmission of a finite array of $N \times N$ nanoblocks with L = 555 nm, H = 695 nm, and P = 736 nm for varying numbers of repetitions N in the array. **b**, Maximum electric field enhancement in the central nanoblock of the finite array with varying number of repetitions. **c**, Quality factor of the transmitted light of the finite array of nanoblocks with varying number of repetitions. The dashed lines in (**b**) and (**c**) show the field enhancement and quality factor of the periodic array for comparison. Beyond N = 10 the response of the finite array is similar to the periodic case.

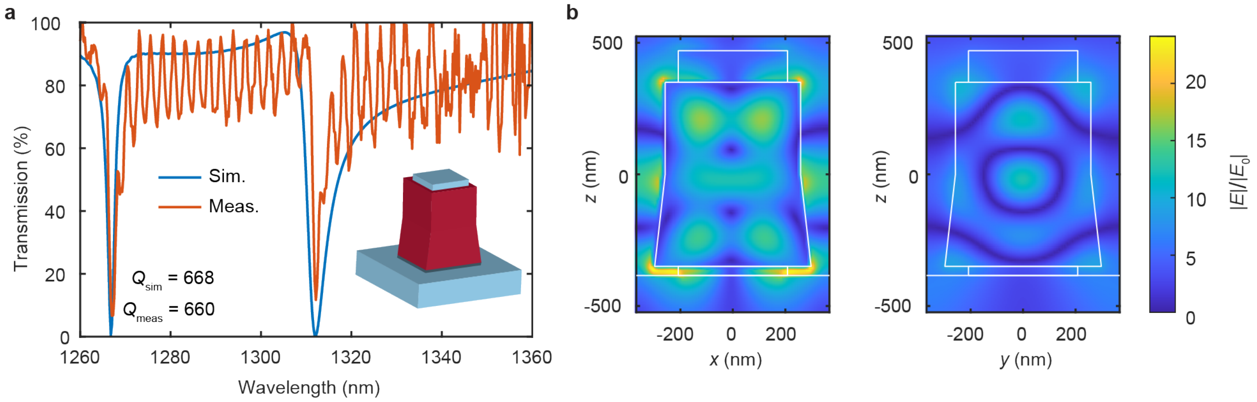

Supplementary Figure 2 | Comparison of experiment to simulation. a, Measured transmission (Meas.) of a nanoblock array with L = 567 nm, H = 695 nm, and P = 736 nm and simulated transmission (Sim.) with modifications in the geometry to account for fabrication imperfections. In the simulation, the length of the nanoblock is L = 516 nm, the side wall tilt α = 12.9°, the height is H = 699 nm, the length and height of the remaining SiO_2 hard mask are L_{SiO2} = 416 nm and H_{SiO2} = 120 nm, and the undercut is d_u = 35 nm deep and 65 nm wide. **b**, Simulated electric field amplitude profiles in an x-z and y-z cross sections of a periodic amorphous silicon nanoblock array on a glass substrate at λ = 1267 nm, with modification in the geometry to account for fabrication imperfections. The similarity to Supplementary Fig. 10 shows that the same ED/EO modes are being measured. The modeled fabrication imperfections include tilted side walls, an undercut below the nanoblock and a residual SiO_2 hard mask as indicated by the white solid lines in (**b**). The simulated and measured quality factors of Q_{sim} = 660 and Q_{meas} = 668 are in good agreement.

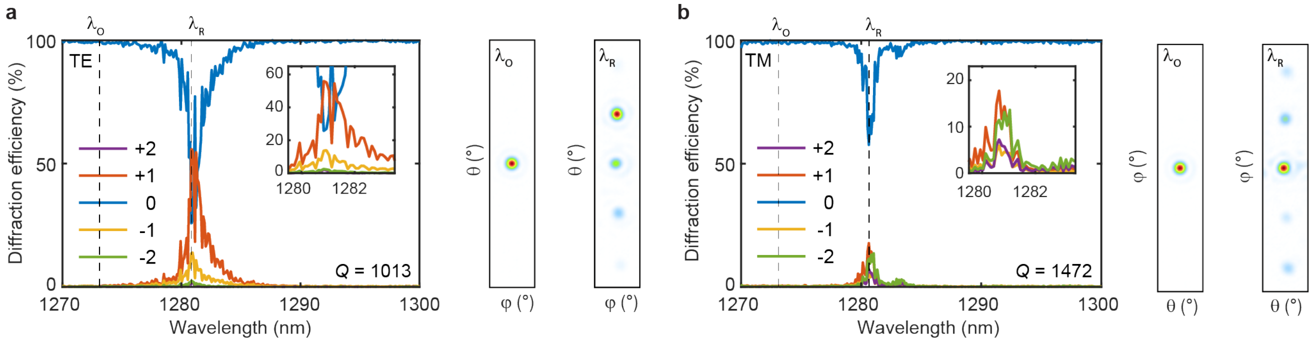

Supplementary Figure 3 | High quality factor beam deflection to θ = 25.8° and φ = 25.8°. a, b, Experimentally measured diffraction efficiencies of the -2 (green), -1 (yellow), 0 (blue), +1 (red) and +2 (purple) diffraction orders and Fourier plane images of a metasurface showing (**a**) TE deflection of *x*-polarized light along the *y* direction and (**b**) TM deflection of *x*-polarized light along the *x* direction. The desired diffraction order is +1, with θ = 25.8° and φ = 25.8° respectively. On resonance, λ_R = 1280.8 nm, a diffraction efficiency of 55.9% and 17.7% is attained for the TE and TM mode, respectively. Off-resonance, λ_O = 1273 nm, 99.6% and 99.6% of the transmitted light remains in the surface normal direction. The insets show a zoomed in region of the plots.

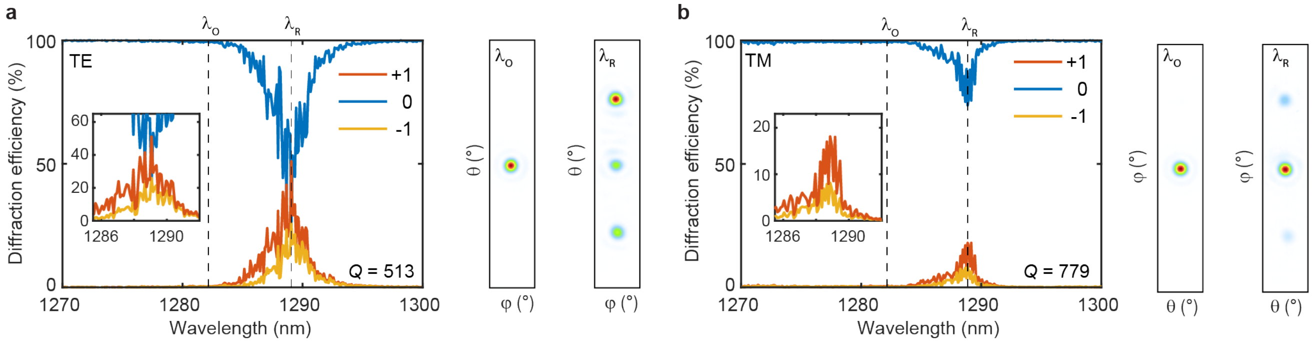

Supplementary Figure 4 | High quality factor beam deflection to θ = 35.7° and φ = 35.7°. a, b, Experimentally measured diffraction efficiencies of the -1 (yellow), 0 (blue) and +1 (red) diffraction orders and Fourier plane images of a metasurface showing (**a**) TE deflection of *x*-polarized light along the *y* direction and (**b**) TM deflection of *x*-polarized light along the *x* direction. The desired diffraction order is +1, with θ = 35.7° and φ = 35.7° respectively. On resonance, λ_R = 1289 nm, a diffraction efficiency of 51% and 18.1% is attained for the TE and TM mode, respectively. Off-resonance, λ_O = 1282 nm, 99.4% and 50.98% of the transmitted light remains in the surface normal direction. The insets show a zoomed in region of the plots.

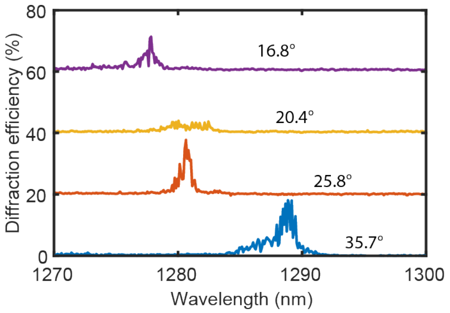

Supplementary Figure 5 | Spectral diffraction efficiency for TM deflection at different angles. Measured spectral diffraction efficiency of TM light deflection with varying deflection angle from the same structures as shown in Fig. 3e for TE light deflection. The measured curves are shifted by 20% from each other for better visibility.

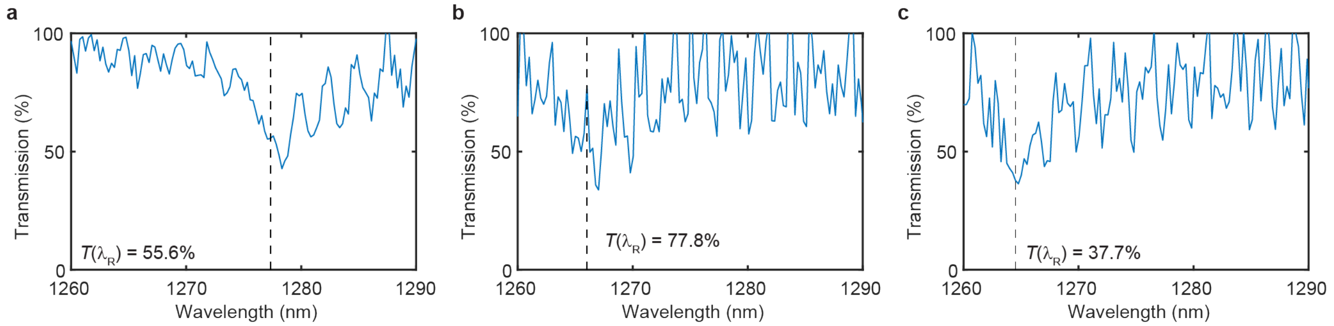

Supplementary Figure 6 | Measured transmission of metalenses. a, Measured transmittance of the metalens from Fig. 4a-e. The transmission on resonance is 55.6%. **b,** Measured transmittance of the metalens from Supplementary Fig. 7a. The transmission on resonance is 77.8%. **c,** Measured transmittance of the metalens from Supplementary Fig. 7b. The transmission on resonance is 37.7%. The resonance wavelength is the wavelength where the focusing efficiency is maximized.

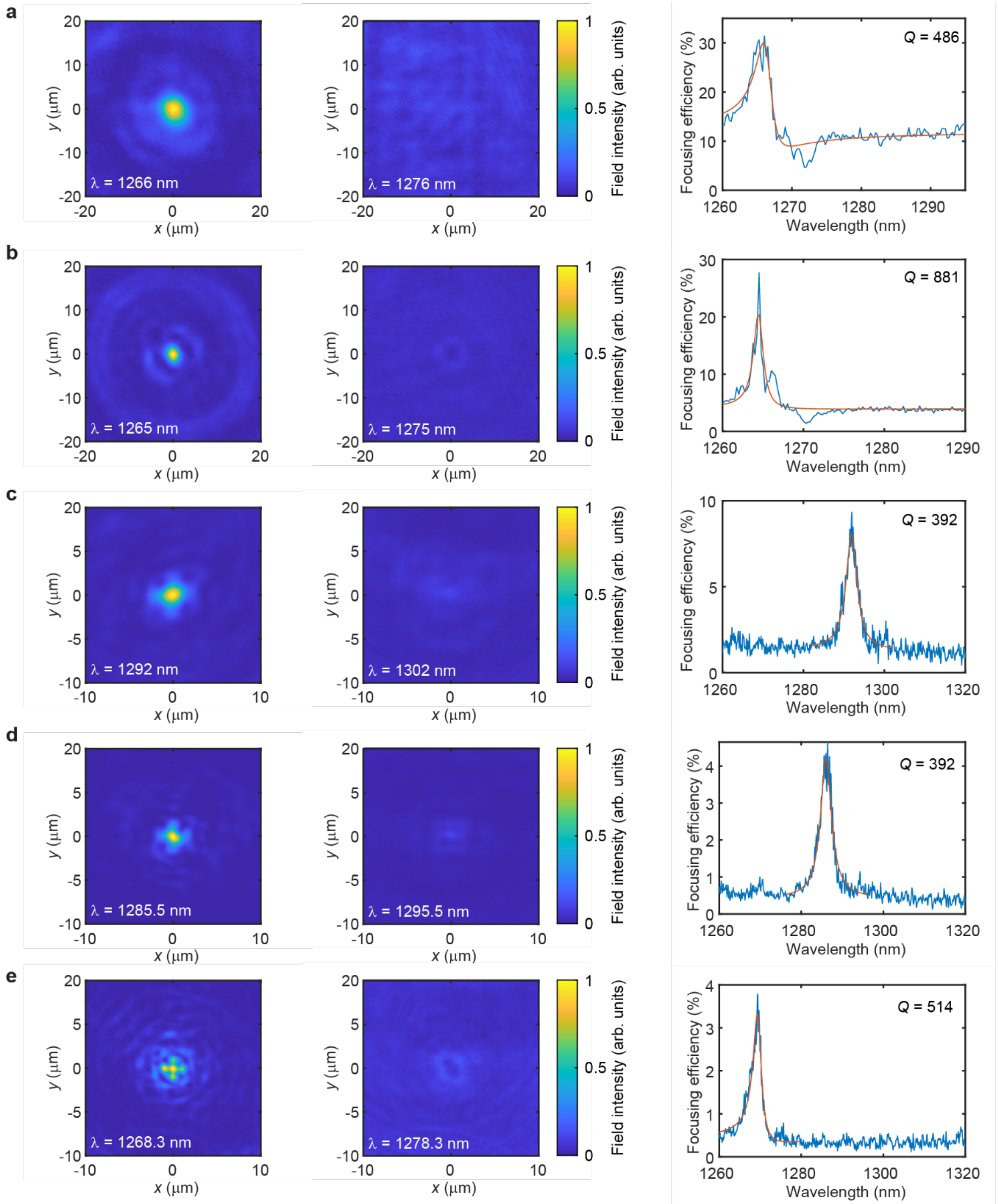

Supplementary Figure 7 | Metalenses with high quality factor. a-e, Measured field intensity at the focal plane on resonance and off resonance, and the measured spectral focusing efficiency of metalenses with numerical aperture of (**a**) 0.1 (**b**) 0.18 (**c**) 0.4 (**d**) 0.6 (**e**) 0.8. The respective resonance wavelengths and determined quality factors are indicated on the panels. The blue line shows the measured focusing efficiency and the red line shows a Fano fit to the data.

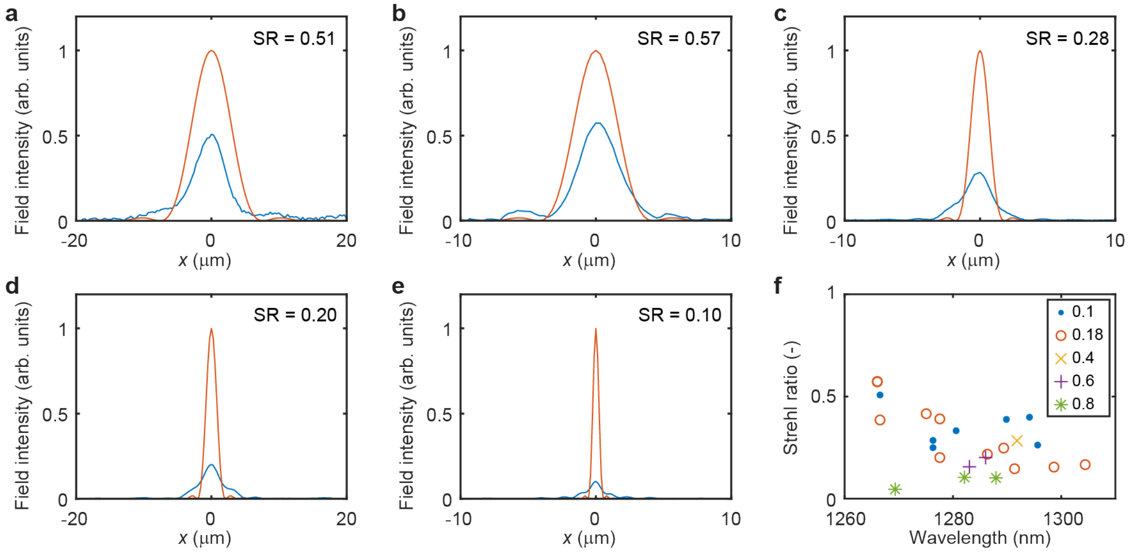

Supplementary Figure 8 | Strehl ratio calculations of characterized metalenses. a-e, Measured field intensity profile at the focal plane on resonance of high-Q metalenses (blue) and an airy disk function (red) with the same numerical aperture for comparison. **f,** Calculated Strehl ratios for the characterized metalenses with numerical apertures of 0.1, 0.18, 0.4, 0.6 and 0.8 and different resonance wavelengths. The Strehl ratio (SR) is calculated by integrating the intensity in the focal plane around the focal spot within a radius of eight times the diffraction-limited airy disk radius.

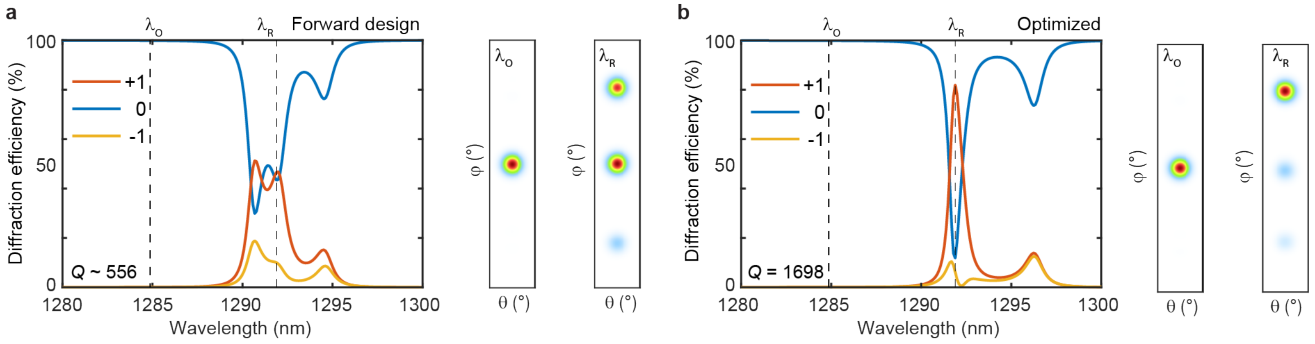

Supplementary Figure 9 | Numerical optimization of high-quality factor TM beam deflection to φ = 35.8°. a, b, Simulated diffraction efficiencies of the -1 (yellow), 0 (blue) and +1 (red) diffraction orders and Fourier plane images of a metasurface showing TM deflection of *x*-polarized light along the *x* direction for (**a**) a forward design structure and (**b**) an optimized structure using a particle swarm optimization. The desired diffraction order is +1, with φ = 35.8°. On resonance, λ_R = 1291.9 nm, a diffraction efficiency of 46.5% and 81.9% is attained for the forward design and the optimized design, respectively. The design includes 3 nanoblocks per Fresnel zone and nanoblock side lengths are [553.9, 554.9, 557] nm and [554.8, 554.9, 558] nm for the forward design and

optimized design respectively. For the optimization the rod lengths L_1 and L_3 are varied and L_2 is fixed. Additionally, the smallest mesh refinement is set to 10 nm, to reduce the computational cost of the optimization.

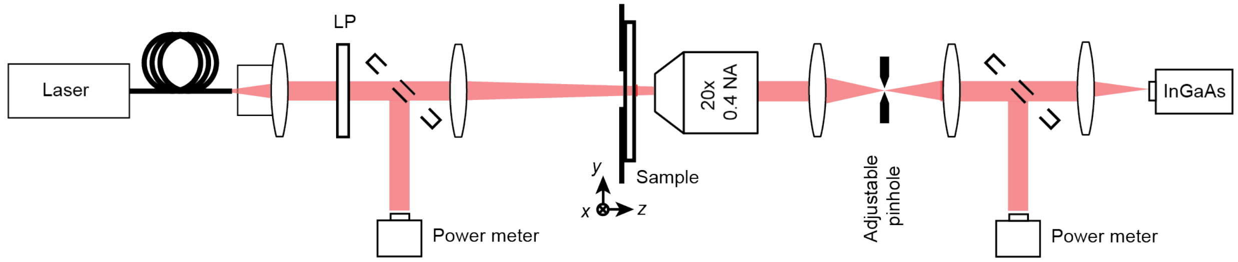

Supplementary Figure 10 | Experimental set-up. The fabricated samples are illuminated in transmission with loosely focused light from a tunable diode laser. The transmitted light is collected by an imaging objective (20x, 0.4 NA) and projected onto a InGaAs IR camera through a set of lenses. With a flip mirror the transmitted light can be either sent to the camera or to a power meter. In the detection path an image plane is formed with an adjustable iris to limit the area that is projected on the camera or power meter. For Fourier plane imaging a 0.9 NA objective lens is used, and a Fourier plane is formed on the camera sensor by exchanging the lens before the camera to a different focal length. A linear polarizer (LP) is used to set the incident light polarization.

Supplementary Tables

Figure panel	λ_R (nm)	θ (°)	Phase gradient (rad/μm)	Fresnel Zones per surface	Nanoblocks per Fresnel Zone	Designed nanoblock lengths (nm)
3a, b, c, d	1295	26	2.134	51	4	[587.1, 588.6, 588.8, 591.5]
3c, d	1272.5	25.6	2.134	51	4	[569.1, 570.6, 570.8, 573.5]
3c, d, e, f	1280.8	25.8	2.134	51	4	[575.1, 576.6, 576.8, 579.5]
3c, d	1306.3	26.3	2.134	51	4	[603.1, 604.6, 604.8, 607.5]
3c, d	1319.4	26.6	2.134	51	4	[613.1, 614.6, 614.8, 617.5]
3e, f	1277.7	16.8	1.423	34	6	[574.2, 576.2, 576.4, 576.6, 577.1, 581.4]
3e, f	1281.2	20.4	1.707	40	5	[574.6, 576.3, 576.5, 576.7, 580.8]
3e, f	1289	35.7	2.846	68	3	[575.6, 576.6, 578.7]

Supplementary Table 1 | Design parameters of beam deflector metasurfaces. Parameters are given for all the beam deflector metasurfaces in Fig. 3. The fabricated metasurface size is 150 μm x 150 μm. The periodicity of the nanoblocks is P = 736 nm.

Lens diameter (μm)	Focal length (μm)	Numerical aperture	Fresnel Zones per surface	Nanoblocks per Fresnel Zone
100	495	0.1	2	20-48
100	274	0.18	4	9-36
100	114.6	0.4	4	5-24
100	66.6	0.6	13	3-18
100	37.5	0.8	39	2-14

Supplementary Table 2 | Design parameters of metalenses. Design parameters are given for the metalenses in Fig. 4 and Supplementary Fig. S7. The periodicity of the nanoblocks is P = 736 nm. The phase distribution is parabolic and the nanorod lengths are chosen according to Fig. 1c. A constant offset is equally added to all nanoblocks of a metalens, resulting in the variation of the resonance wavelengths in Figure 4f.

Supplementary Note 1: Mode profiles and multipole expansion

To gain an understanding of the optical modes supported by the metasurface unit cells we perform finite difference time domain simulations. Supplementary Fig. 11 shows the electric and magnetic field profiles on resonance in a periodic array of amorphous silicon nanoblocks with the size corresponding to the simulation shown in Fig. 1b. The electric and magnetic field are enhanced by a factor of 28 and 46, respectively.

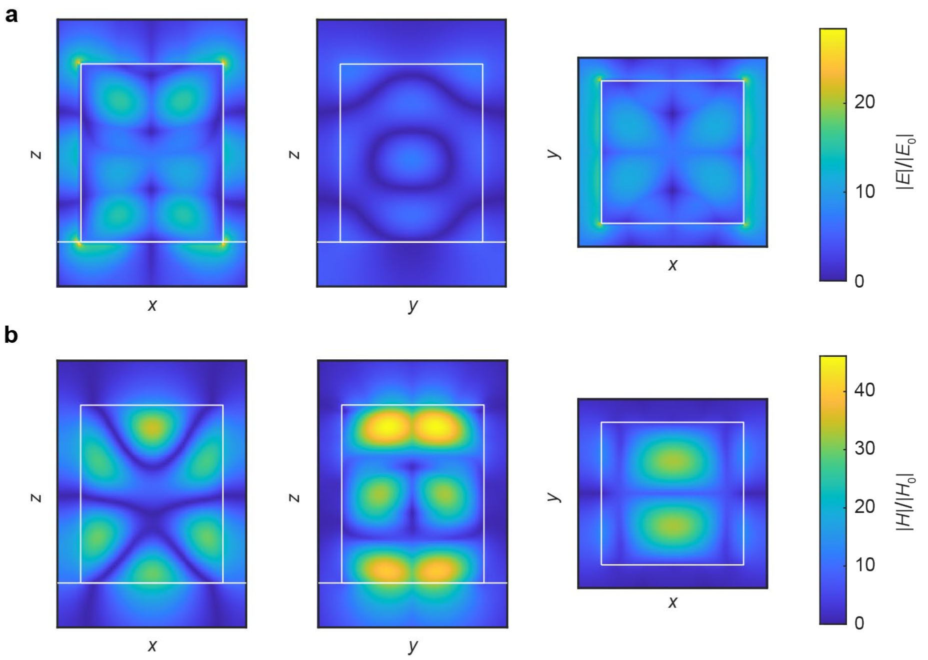

Supplementary Figure 11 | Profiles of electric and magnetic field amplitude in a periodic array. Simulated electric (**a**) and magnetic (**b**) field amplitude in an amorphous silicon nanoblock in a periodic array on a glass substrate with L = 555 nm, H = 695 nm, and P = 736 nm at a wavelength λ = 1288 nm. The illumination is incident along the positive z direction with the polarization along the x direction. Cross sections are shown for y = 0, x = 0 and z = H/2. The electric and magnetic field are normalized by the incident field amplitude E_0 and H_0, respectively. The magnetic field profile closely resembles the profile of an electric octupole mode in an isolated sphere as shown in Supplementary Fig. 15.

To understand the origin of the resonance mode and the high quality factor we perform a multipole expansion[1]. Supplementary Fig. 12 illustrates the different resonant components of the scattering cross section of an individual nanoblock in free space as calculated from a multipole expansion. Clear resonant features are observed such as the magnetic dipole (MD), electric dipole

(ED), magnetic quadrupole (MQ) and electric octupole (EO). There is good agreement between the scattering cross section calculated from the multipole expansion and the corresponding FDTD calculation. Of all the resonant modes the EO at λ = 1.16 μm is specifically in the vicinity of the operation wavelength of the metasurface. The corresponding electric and magnetic field profiles of the EO at λ = 1.16 μm of a single nanoblock in free space are shown in Supplementary Fig. 13 as simulated from FDTD simulations. For these simulations, a total-field scattered-field source and perfectly matched layer boundary conditions were used. In the magnetic field profiles the resemblance to the field profiles of the periodic array in Supplementary Fig. 11 is evident. The linewidth of the EO is approximately 9 nm, suggesting that in a periodic array the near-field coupling of the neighboring nanoblocks further narrows the resonance, something that is also observed in low-order Mie-resonant metasurfaces[2]. We expect that for our metasurface, the influence of the substrate and the neighboring elements in the array red shift the EO resonance to λ = 1.288 μm.

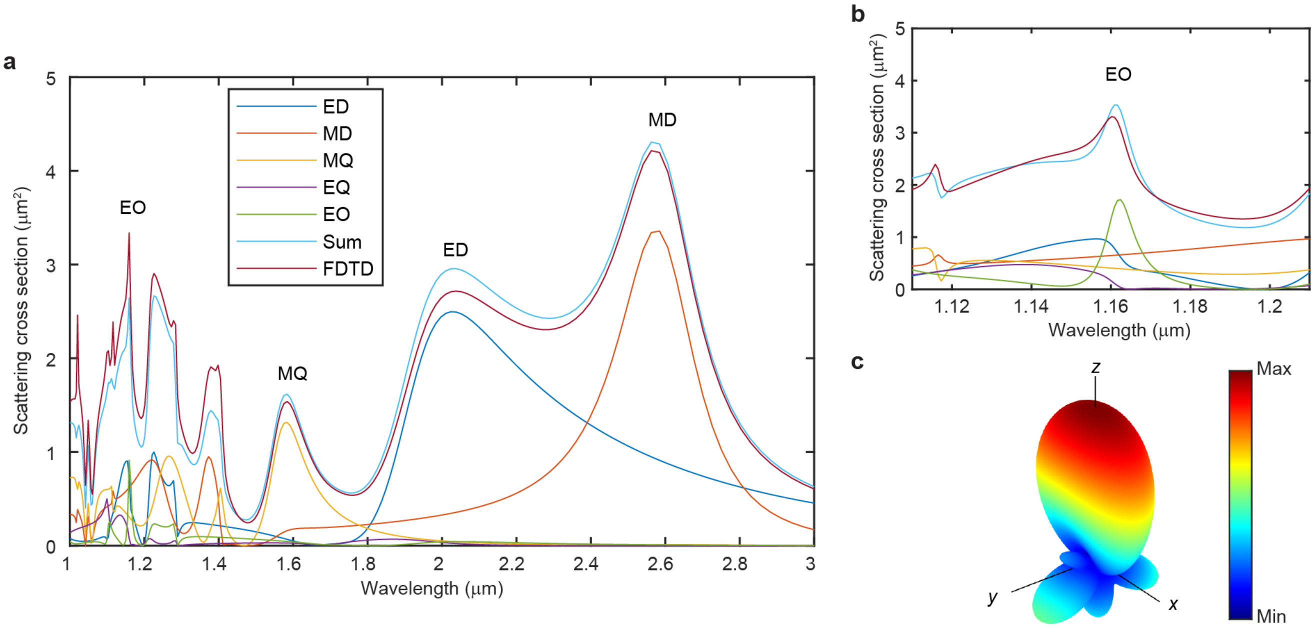

Supplementary Figure 12 | Multipole expansion of an isolated nanoblock. a, Scattering cross section of a single amorphous silicon nanoblock in free space with L = 555 nm and H = 695 nm calculated using the multipole expansion method[1]. For comparison, the sum of the multipoles and the scattering cross section as calculated by FDTD is shown. **b**, A zoomed in view of (**a**) over the wavelength range of the electric octupole mode. **c**, Radiation pattern of an isolated nanoblock in free space at the EO resonant wavelength λ = 1.16 μm.

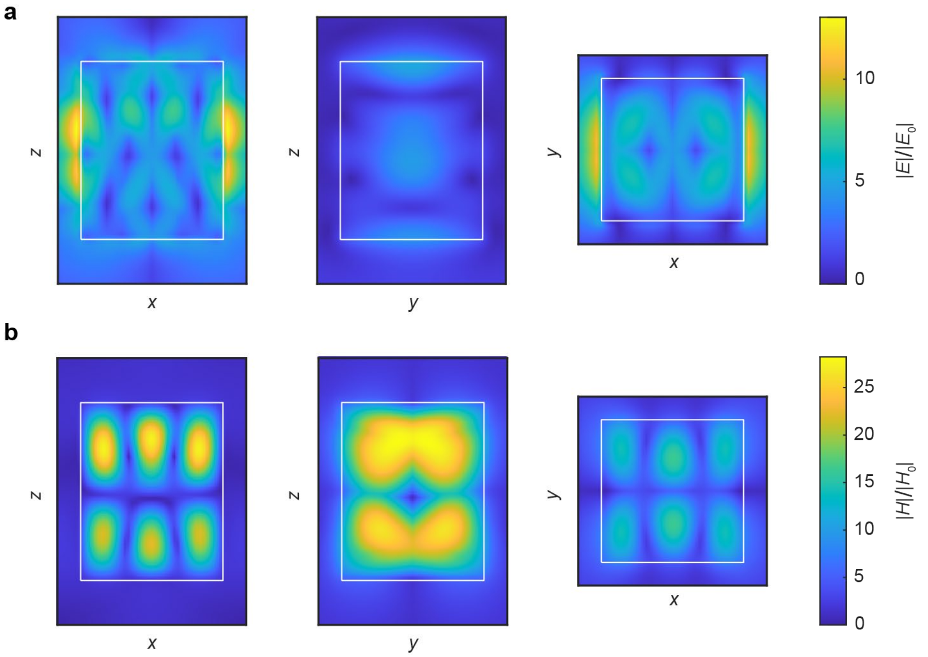

Supplementary Figure 13 | Profiles of electric and magnetic field amplitude of an isolated nanoblock. Simulated electric (**a**) and magnetic (**b**) field intensity in an amorphous silicon nanoblock in free space with L = 555 nm and H = 695 nm at a wavelength λ = 1.16 μm. The illumination is along the positive z direction with the polarization along the x direction. Cross sections are shown for y = 0, x = 0 and z = H/2.

To account for the neighboring effect between different nanoblocks, we adopt the formalism by Savinov et al.[3] to calculate the reflected field amplitude and phase of a periodic array of nanoblocks from the multipoles. Supplementary Fig. 14a shows the contributions of the different multipoles towards the reflected field amplitude. A strong reflectance peak is observed at the resonance wavelength of our metasurface. The main contributions to the reflected field amplitude are due to the spectrally overlapped electric dipole and electric octupole modes. This spectral overlapping of the ED and EO, and their interference with the transmitted light, results in a narrow high quality factor resonance with a vanishing transmission on resonance. The phase of each of the multipole terms in the reflected field is shown in Supplementary Fig. 14b. On resonance the ED and EO are in phase and of equal magnitude leading to a generalized Kerker effect. We note that although the formalism by Savinov et al.[3] was derived for periodic arrays of scatterers without a substrate, a qualitative analysis can still be made in the presence of a substrate[4]. We further also confirmed the presence of the EO/ED mode without a substrate and similar behavior in the reflection amplitude and phase was obtained as for the case with a substrate shown here.

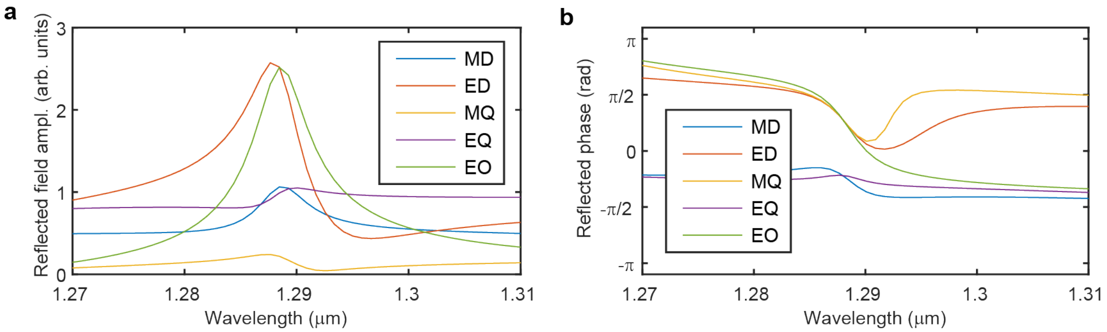

Supplementary Figure 14 | Multipole contributions of the periodic metasurface. Multipole contributions of the to the reflected far field electric field amplitude (ampl.) (**a**) and the corresponding phase of each component (**b**) for a periodic amorphous silicon nanoblock array on a glass substrate with *L* = 555 nm, *H* = 695 nm, and *P* = 736 nm.

For comparison with the field profiles of the nanoblocks, in Supplementary Fig. 15 we illustrate the electric and magnetic field profiles of an EO mode in a spherical nanoparticle in air as calculated from FDTD simulations. Here, the radius of the particle is 400 nm, and the index of refraction is set to $n = 4$. In this geometry, the EO is induced at a wavelength $\lambda = 1475$ nm. Notably, the magnetic field of the EO closely resembles the magnetic field in the metasurface for the periodic array of nanoblocks on a glass substrate shown in Supplementary Fig. 11.

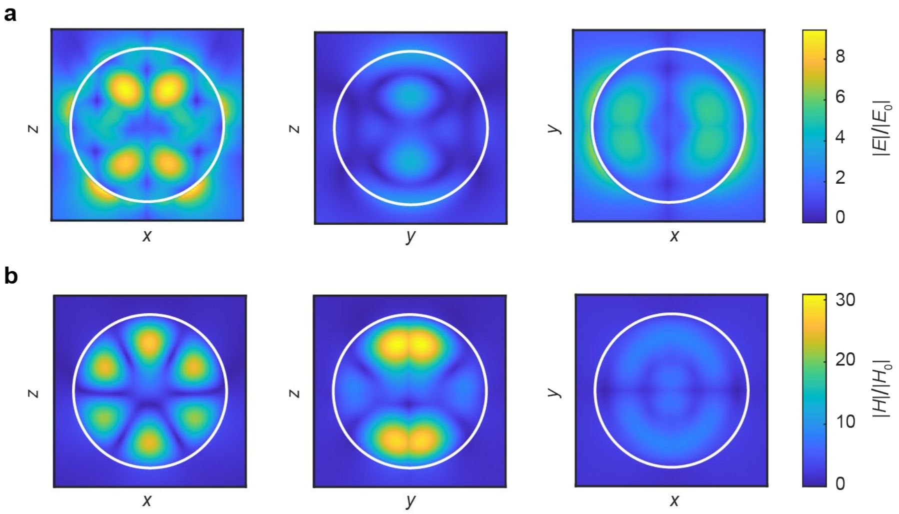

Supplementary Figure 15 | Profiles of electric and magnetic field amplitude of an isolated nanosphere. Simulated electric (**a**) and magnetic (**b**) field intensity in a nanosphere in free space with radius 400 nm and refractive index $n = 4$ at a wavelength $\lambda = 1475$ μm. The illumination is along the positive *z* direction with the polarization along the *x* direction. Cross sections are shown for $y = 0$, $x = 0$ and $z = 0$.

Supplementary Note 2: Distinction from supercavity modes

We further analyze the properties of the ED/EO mode and the light scattering of our structure in the context of local supercavity modes reported in isolated nanoparticles nanoparticles[5,6]. We record the total light scattered by a metasurface with uniformly sized nanoblocks using finite difference time domain simulations. To this end, we employ a total field scattered field light source with periodic boundary conditions. Supplementary Fig. 16a illustrates the total scattered light for varying side length *L* of the nanoblocks and a fixed nanoblock height. Supplementary Fig. 16b and c illustrate the quality factor and the Fano parameter for varying *L* as determined from a Fano fit to the total scattered light. The data for the structure illustrated in Fig. 1b, *L* = 555 nm, is highlighted in bold. We observe a linear shift of the ED/EO resonance wavelength with a change nanoblock aspect ratio. As compared to local supercavity modes, the ED/EO mode does not undergo a mode splitting and anti-crossing. Furthermore, we observe a slow variation of the quality factor with variation of the nanoblock side length and a Fano parameter within the range of -0.8 to -0.4. Conversely, for a supercavity mode the quality factor of the scattered light varies rapidly as a function of a geometric parameter and the Fano parameter diverges to infinity at the supercavity condition. Additionally, reports on supercavity modes to date observe scattering from an isolated nanoparticle, whereas the nanoblocks in our structure are arranged in a sub-diffractive array, where there is significant coupling between neighboring elements. This suggests that our concept of a higher order Mie resonant metasurface is different from the previously reported localized supercavity modes.

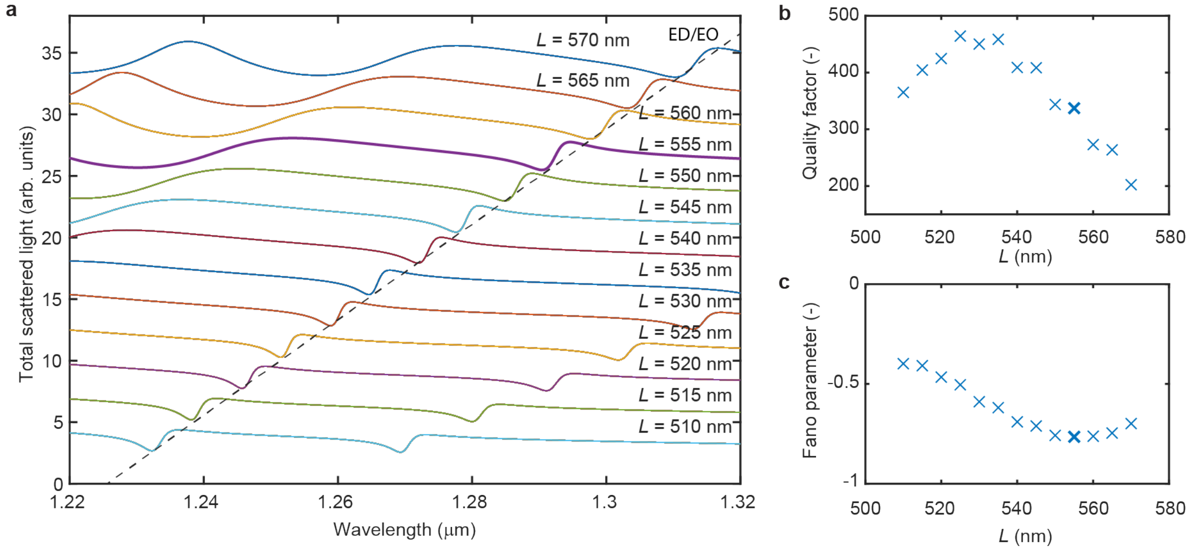

Supplementary Figure 16 | Light scattering from ED/EO metasurfaces. a, Simulated total scattered light from the higher order Mie resonant metasurfaces with *H* = 695 nm and *P* = 736 nm and *L* = 510–570 nm. Curves are displaced vertically by 2.5 for better visibility. Quality factors (**b**) and Fano parameters (**c**) extracted from the total scattered light in (**a**) with a Fano fit.

Supplementary Note 3: Mode localization

To investigate the localization of the ED/EO mode we perform FDTD simulations where we excite the mode in a single resonator within a finite sized array (11 × 11 repetitions). The mode is resonantly excited with an electric dipole source at the center of the resonator polarized along the *x* direction. We record the electric field components around the excited resonator over an area of 5 × 5 repetitions. The electric field is recorded using an apodization time to suppress the initial field components of the dipole excitation. Supplementary Fig. 17 illustrates the electric field intensity in different cross sections around the excited central resonator. The electric field pattern in the central resonator is identical to the one of the resonators in the periodic array (see Supplementary Fig. 11). As a measure of mode localization, we numerically evaluate the ED/EO mode volume according to

$$V = \int \frac{\varepsilon(\mathbf{r}) \cdot |\mathbf{E}(\mathbf{r})|^2}{\max\left(|\mathbf{E}(\mathbf{r})|^2\right)} d\mathbf{r}^3, \quad (1)$$

where **E** denotes the electric field at position **r**. In our calculation, we obtain a mode volume of 1.8 μm^3. The volume of a unit cell is estimated to 0.736 μm × 0.736 μm × 0.895 μm ≈ 0.5 μm^3, with an added 0.2 μm to the height as an approximate extent of the evanescent component in *z*. Comparing this volume to the mode volume, suggests that the mode is localized around a single resonator and its nearest neighbors. This can also be observed in Supplementary Fig. 17.

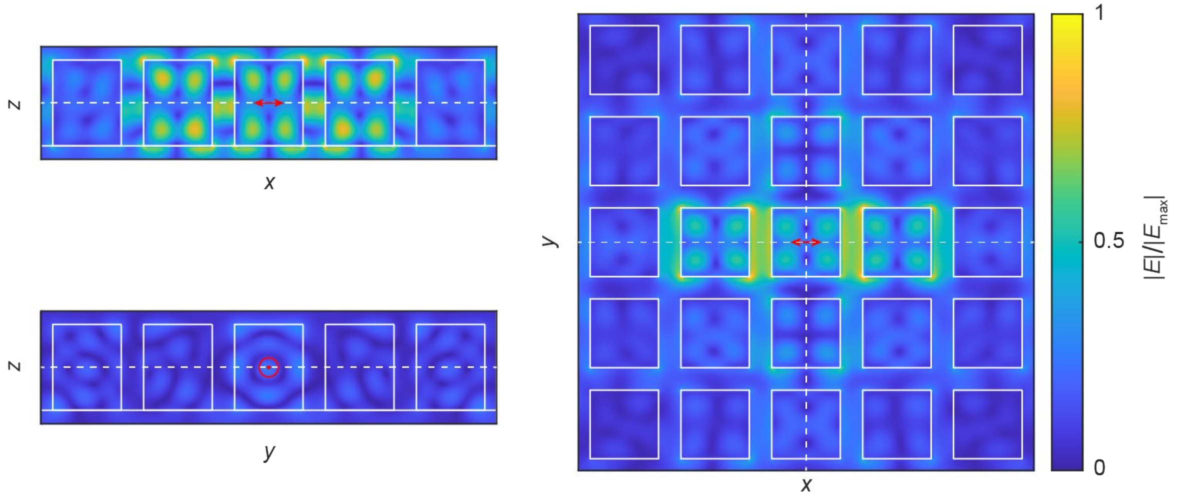

Supplementary Figure 17 | Dipole excitation of EO/ED mode. Electric field amplitude profiles in an *x-z* cross section for *y* = 0 nm, in a *y-z* cross section for *x* = 0 nm and in a *x-y* cross section for z = *H*/2 as indicated by the white dashed lines. The field intensity color scale for all the panels is identical. The red arrow indicates the

position of the electric dipole that was used to excite the mode. The geometrical parameters of the structure are *L* = 555 nm, *H* = 695 nm, and *P* = 736 nm.

In a separate analysis, we study the reflection of a finite-sized array of uniformly sized nanoblocks. We write the reflection of a uniform *N* × *N* array of nanoblocks as a summation of the backward scattering $\sigma_{u,L}$ of each nanoblock with length *L*

$$R_{u,L} = \gamma \sum_{n}^{N^2} \sigma_{u,L} = \gamma \cdot N^2 \cdot \sigma_{u,L} \,, \tag{2}$$

where γ is a lumped parameter accounting for the finite size and light coupling to each nanoblock. Next, we introduce a perturbation in the array, by modifying the length of one nanoblock in the array to *L* + *dL.* We assume the hypothesis that the scattering of each nanoblock is independent. For this case, the total reflection of the perturbed array can be obtained by

$$R_{p,L+dL} = \gamma \sum_{n}^{N^2-1} \sigma_{u,L} + \gamma \cdot \sigma_{p,L+dL} = \gamma \cdot (N^2 - 1) \cdot \sigma_{u,L} + \gamma \cdot \sigma_{p,L+dL}, \tag{3}$$

where $\sigma_{p,L+dL}$ represents the backward scattering of a single nanoblock with length *L* + *dL* in a perturbed array. Following this logic, by combining Eq. (2) and (3), we can retrieve the difference in backward scattering between an individual nanoblock of length *L* and one of length *L* + *dL* as

$$\Delta\sigma_{pu} = \sigma_{p,L+dL} - \sigma_{u,L} = \frac{R_{p,L+dL} - R_{u,L}}{\gamma}. \tag{4}$$

This difference can then be determined from FDTD simulations. Two simulations are preformed, one simulation with an identical array of nanoblocks with *L* = 555 nm, and another simulation of a perturbed array, where the central nanoblock has a length of *L* + *dL* = 558 nm and the remaining nanoblocks *L* = 555 nm. Supplementary Fig. 18a illustrates the recorded reflection for both cases. There is only a very small difference between the overall reflection of the uniform and the perturbed array. This shows that the resonance and the quality factor of the mode are robust with respect to the perturbation. With the use of Eq. (4) we can now calculate the difference in backward scattering as shown in Supplementary Fig. 18b. To validate our hypothesis of independent nanoblocks the same difference in backward scattering is calculated from the reflection of two uniform, unperturbed arrays by using Eq. (2),

$$\Delta\sigma_{uu} = \sigma_{u,L+dL} - \sigma_{u,L} = \frac{R_{u,L+dL} - R_{u,L}}{N^2\gamma}. \tag{5}$$

Supplementary Fig. 18b shows the calculated difference in backward scattering $\Delta\sigma_{uu}$ and $\Delta\sigma_{pu}$. A positive value of $\Delta\sigma_{pu}$ at wavelength larger than the resonance wavelength corresponds to a redshift of the scattering of the nanorod with length $L + dL$, as expected from the uniform array calculation. The differences in scattering, $\Delta\sigma_{uu}$ and $\Delta\sigma_{pu}$, show good qualitative agreement. This indicates that, to a first approximation, the scatterers can be treated of as independent from each other, hence allowing for a local control of the scattering properties of each nanoblock. As such, the nanoblocks can be approximated as effective local point scatterers. The difference in scaling between $\Delta\sigma_{uu}$ and $\Delta\sigma_{up}$ is likely due to finite size effects related to the scattering in the perturbed array and nearest neighbor interaction.

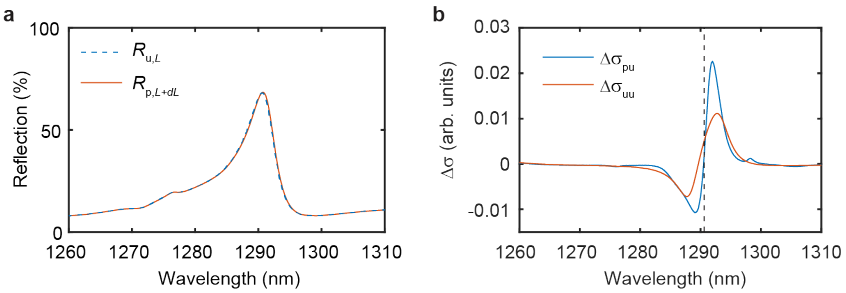

Supplementary Figure 18 | Reflection and scattering analysis. a, Simulated reflection spectrum of a finite sized array (9 × 9) of amorphous silicon nanoblocks on a glass substrate of a uniform array with L = 555 nm, and a perturbed array with the central resonator of length $L+ dL$ = 558 nm and the remaining resonators with length L = 555 nm. In both cases, H = 695 nm, and P = 736 nm. **b**, Difference in the backward scattering as calculated by Eq. (4) or (5). The dashed line highlights the resonance wavelength of the uniform array with L = 555 nm.

Supplementary Note 4: On the non-locality of asymmetry induced q-BIC modes

We draw a comparison of our work to the asymmetry-induced q-BIC that have been thoroughly investigated in the literature[7–9], specifically focusing on mode localization and resonance-based beam steering. The high-quality factor resonance observed in a q-BIC structure can also be used for modulating the phase of the transmitted light[10]. To illustrate this, we simulate the structure reported by Campione et al.[7] with geometric parameters adapted to shift the resonance to the near infrared spectral range. A narrow resonance is obtained in transmission with Q = 651 as illustrated in Supplementary Fig. 19a. Similar to the higher-order Mie-resonant metasurface presented here, modifying the length L of the q-BIC resonator, can be used to spectrally shift the resonance and imprint a phase shift covering close to the entire phase range of 0-2π (see Supplementary Fig. 19b). Based on the relationship between phase and length L, a hypothetical beam deflection metasurface can be designed. Here, we use a metasurface with three notched nanoblocks per Fresnel zone, with lengths L_1, L_2, and L_3, designed to deflect light to 38.2° at a wavelength of 1327 nm. As shown in Supplementary Fig. 19c and d, for both TE and TM Deflection, 100% of the transmitted light remains in the normal direction, and no light is coupled to the angles ±38.2°. This is due to the non-locality of asymmetry-induced q-BIC modes, which inherently prohibits beam steering. This represents a substantial difference to the higher-order Mie-resonant metasurfaces demonstrated here, where the locality of the mode enables wavefront manipulation in two dimensions. While conventional design approaches fail to achieve wavefront manipulation, notably, topological optimization can be employed to realize high-Q metagratings for 1D beam deflection with symmetry protected q-BIC structures[11].

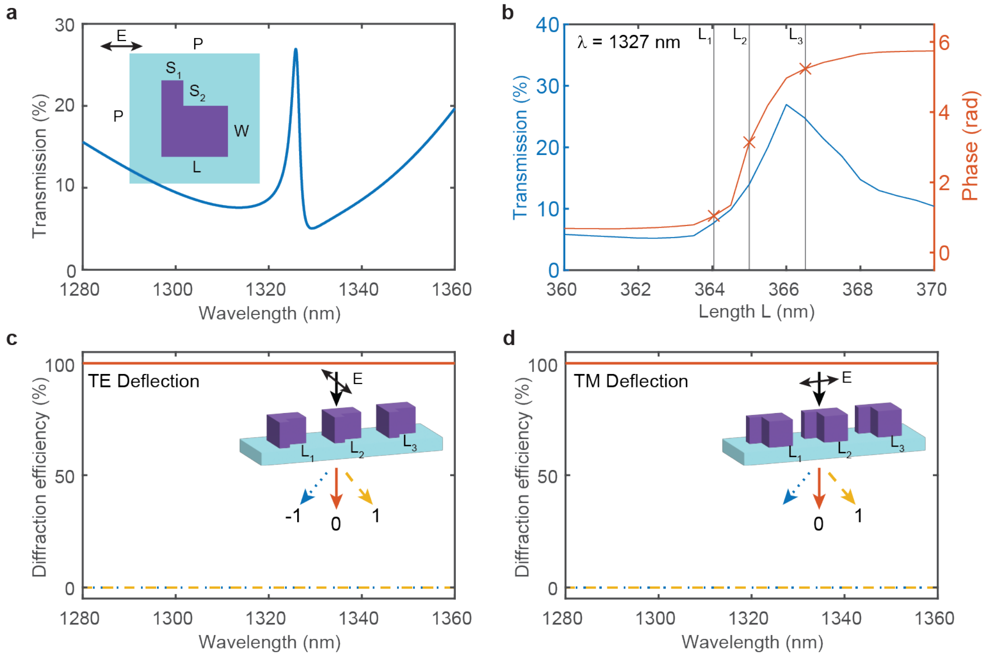

Supplementary Figure 19 | Non-locality of asymmetry induced q-BIC prohibits 2D resonance-based wavefront manipulation. a, Simulated transmission of a periodic array of asymmetry induced q-BIC structure based on the work of Campione et al.[7]. The resonator consists of amorphous silicon and the geometric parameters are scaled to the near infrared range and correspond to L = 365 nm, W = 280 nm, S_1 = 120 nm, S_2 = 140 nm, P = 716 nm, and a height of 326 nm. **b**, Simulated transmission and phase of the transmitted light with varying length L, showing the resonance based tuning of the array transmission. The rod lengths L_1, L_2 and L_3 correspond to the rod lengths of a beam deflection metasurface with three nanorods per Fresnel zone. **c**, Simulated diffraction efficiency of a TE beam deflection metasurface using the nanoblocks with L_1, L_2 and L_3, designed to deflect light to 38.2° at an operating wavelength of 1327 nm. **d**, Simulated diffraction efficiency for a TM beam deflection metasurface using the nanoblocks with L_1, L_2 and L_3, designed to deflect light to 38.2° at an operating wavelength of 1327 nm. Due to the non-locality of the q-BIC mode, in both designs all transmitted light resides in the normal direction, illustrating the inability to perform beam steering with these structures.

Supplementary Note 5: Effect of geometric parameter variation

The variation of geometric parameters has a large effect on the optical properties of high quality factor metasurfaces. To analyze this effect, we simulate a uniform nanoblock array with the geometrical parameters from Supplementary Fig. 2 and examine the effect of changing the nanoblock side length L, height H, side wall tilt angle α and undercut depth d_u. Supplementary Fig. 20 shows the simulated transmission for the variation of these geometrical parameters. Notably, the variation of each parameter manifests itself mainly in a spectral shift of the optical resonance. For the variation of the nanoblock side length (Supplementary Fig. 20a) this shift is most pronounced, with 1.3 nm wavelength per nanometer side length change.

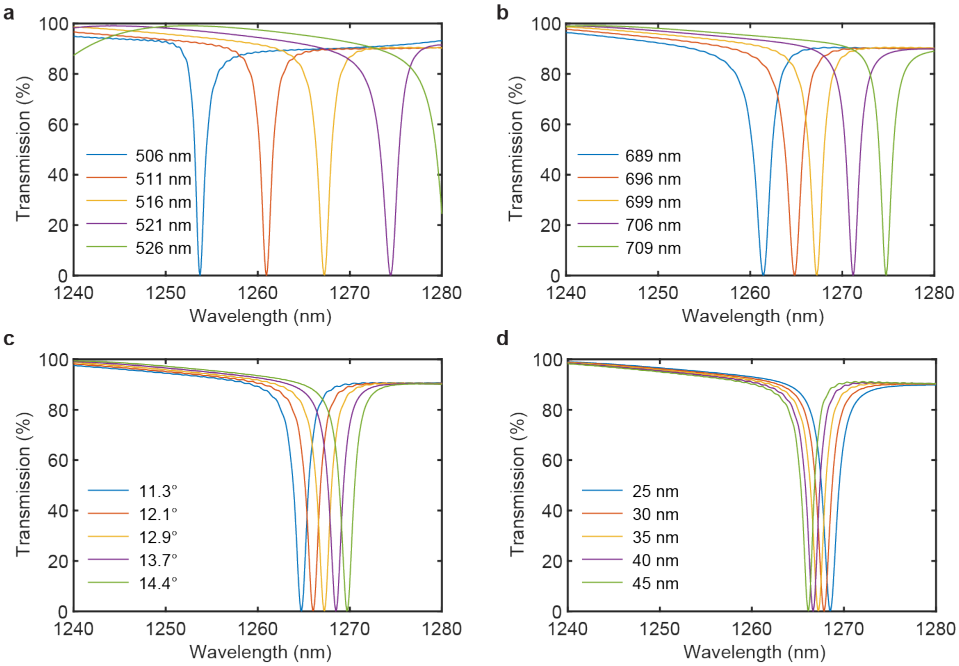

Supplementary Figure 20 | Effect of geometric parameter variation | a, Simulated transmission of a nanoblock array with dimensions shown in Supplementary Fig. 2, P = 736 nm, H = 699 nm, α = 12.9°, a d_u = 35 nm deep and 65 nm wide undercut, and varying rod length L. **b**, Simulated transmission of the structure in (a) with L = 516 nm and varying nanoblock height. **c**, Simulated transmission of the structure in (a) with L = 516 nm and varying nanoblock side wall angle α. **d**, Simulated transmission of the structure in (a) with L = 516 nm and varying undercut of the nanoblock.

In experiment, a non-uniformity of the geometric parameters over the metasurface aperture results in a superposition of many of high-Q scatterers with varying resonant wavelength. Consequently, a variation of L, H, α or d_u, is expected to result in a wider resonance peak of the

surface, hence a reduced quality factor and an increase in the transmission minimum of the surface. To further understand this effect, we numerically model the non-uniformity of our structure by limiting the variation only to the nanoblock side length, since the side length has the largest effect on the spectral resonance shift. We model the non-uniformity by considering a 4 x 4 array of nanoblocks that is periodically repeated and impose a normal distribution of the side length on the 16 nanoblocks in the array. Supplementary Fig. 21 illustrates the calculated transmission for a mean side length of L = 516 nm and varying standard deviation of the normal distribution of 0, 5, 10 and 20 Å. As expected, increasing the structure non-uniformity decreases the quality factor and increases the minimum transmission. Comparing this analysis to the results of Fig. 2, suggests that a standard deviation of 6 Å most closely matches the experimentally observed transmission minimum and quality factor. However, in practice, we expect all parameters L, H, α or d_u to obey a normal distribution with different respective standard deviations. Assuming these parameters affect the optical response independently, a compound standard deviation of the geometry can be approximated as

$$\sigma_{tot}^2 = \sigma_L^2 + \sigma_H^2 + \sigma_\alpha^2 + \sigma_{du}^2. \quad (6)$$

The simulated case in Supplementary Fig. 21, assumes that only σ_L is non-zero. For σ_L = 6 A, any non-uniformity in H, α or d_u will result in a further decrease of the quality factor and an increase of the transmission minimum. This value therefore represents an estimated upper bound, suggesting that the non-uniformity of the side length in our fabricated devices is lower than 6 A.

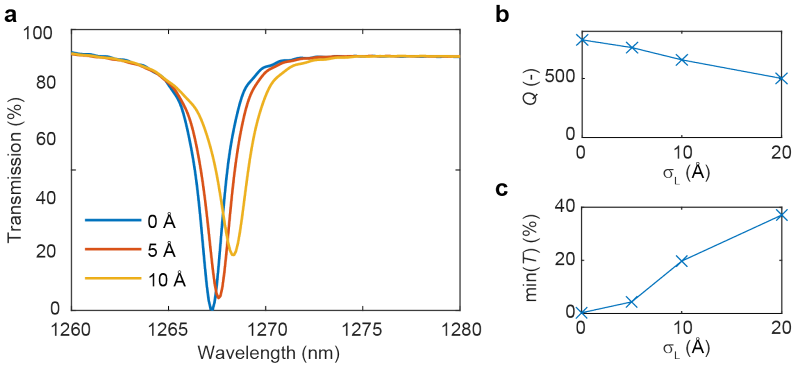

Supplementary Figure 21 | Effect of nanoblock side length variation. a, Simulated transmission of a nanoblock array with randomly varying side length L according to a Gaussian distribution with mean L = 516 nm and varying standard deviation σ_L = 0, 5, 10 Å. The geometry of the structure is in accordance with the geometry in Supplementary Fig. 2, namely a sidewall tilt α = 12.9°, height H = 699 nm, a length and height SiO_2 hard mask

of L_{SiO2} = 416 nm and H_{SiO2} = 120 nm, and a d_u = 35 nm deep and 65 nm wide undercut. **b**, Simulated quality factor of the nanoblock array in (**a**) with varying standard deviation of the Gaussian distribution. **c**, Simulated minimum transmission on resonance of the nanoblock array in (**a**) with varying standard deviation of the Gaussian distribution. The structure dimensions for the simulation are the same as for Supplementary Fig. 2. The FDTD simulation here considers a periodic array (i.e. a periodic repetition) of 4 × 4 nanoblocks, each with a different side length according to a Gaussian distribution.

Supplementary Note 6: Reflective higher-order Mie-resonant metasurfaces

To avoid a strong variation of the scattered electric field amplitude from the metasurface unit cell (see Fig. 1b), we add a gold reflector to the metasurface with an SiO_2 gap between the nanoblocks and the reflector. Supplementary Fig. 22a shows the reflectance and the phase of the reflected light of the metasurface at the ED/EO resonance for L = 520 nm, P = 736 nm, H = 699 nm, and d_{SiO2} = 500 nm. The reflectance remains above 88%, across the resonance. Similar to Fig. 1c, we can adjust the nanoblock side length to set the phase of the reflected light (see Supplementary Fig. 22b). Using this relation of phase vs. side length we can design beam deflectors analogous to the ones demonstrated in Fig. 3. Supplementary Fig. 22c, d and e show the simulated diffraction efficiency of for TE and TM deflection to an angle of 35.2° and the total transmission for both cases. A peak diffraction efficiency of 68% for the TE and 79% for the TM case is attained. At the operation wavelength 1274 nm a total efficiency of light deflection of 65% and 69% is observed for TE and TM deflection, respectively.

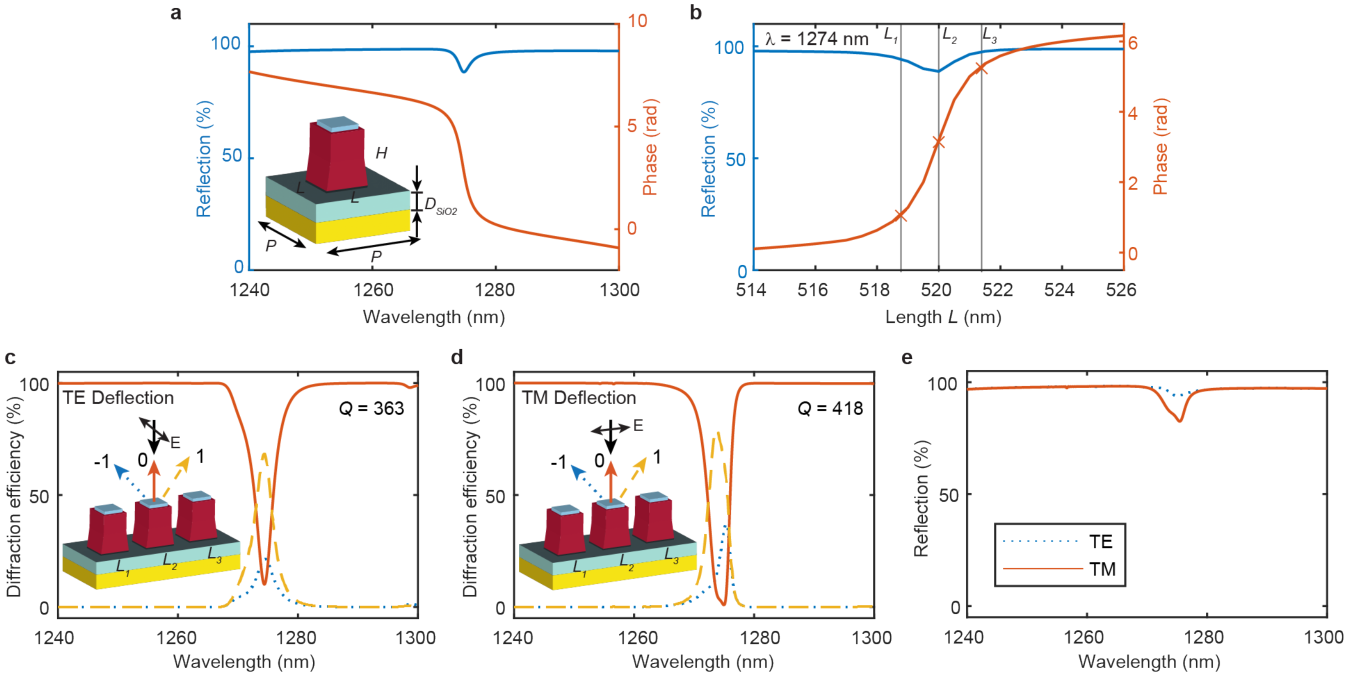

Supplementary Figure 22 | Reflective higher-order Mie-resonant metasurfaces a, Simulated reflectance of a periodic array of nanoblocks on a gold reflector with dimensions to L = 520 nm, H = 699 nm, P = 736 nm, d_{SiO2} = 500 nm, sidewall tilt α = 12.9°, height H = 699 nm, L_{SiO2} = 416 nm and H_{SiO2} = 120 nm, and d_u = 35 nm. **b**, Simulated reflectance and phase of the reflected light with varying length L, showing the resonance based tuning of the phase of the reflected light. The rod lengths L_1, L_2 and L_3 correspond to the rod lengths of a beam deflection

metasurface with three nanoblocks per Fresnel zone. **c**, Simulated diffraction efficiency of a TE beam deflection metasurface using the nanoblocks with L_1, L_2 and L_3, designed to deflect light to 35.2° at an operating wavelength of 1274 nm. **d**, Simulated diffraction efficiency for a TM beam deflection metasurface using the nanoblocks with L_1, L_2 and L_3, designed to deflect light to 35.2° at an operating wavelength of 1274 nm. **e**, Simulated reflection for the TE and TM beam deflection metasurface.

Supplementary Note 7: Comparison to the state of the art

We compare our work to the current state of the art of two dimensional wavefront shaping with high quality factor. The current method is based on geometric phase and the rotation of high quality factor birefringent unit cells, so called non-local metasurfaces[12,13]. The response of these non-local metasurfaces can be described by a bandstructure, with their resonance frequency for an incidence angle θ given by

$$\omega_{res} = \omega_0 + bk^2, \tag{7}$$

where b is the band curvature, $k = k_0 \sin\theta$, k_0 the free space wave vector, and ω_0 is the resonant angular frequency of at k = 0.

Figure 5 compares the state of the art of experimental realizations in terms of quality factor, numerical aperture, and efficiency. Supplementary Table 3 shows a detailed comparison of non-local metasurfaces[12,13] to the present work, focusing on important parameters for wavefront shaping with metasurface optical elements. A discussion of each parameter and the state of the art follows below.

**Performance Metrics**	**Non-local metasurfaces[12,13]**	**Higher-order Mie-resonant metasurfaces (this work)**
**Quality factor**	< 86 for 2D manipulation<br>< 300 for 1D manipulation	< 880 for 2D manipulation<br>< 1492 for 1D manipulation
**Numerical aperture**	0.2, limited to < 0.26	Up to 0.8 or higher
**Overall efficiency**	8%, radial metalens<br>Limited to 25%	24.4 % radial metalens<br>No upper limit known
**Incidence angle dependence / dispersion**	TE: 9 nm / 10°, TM: 70 nm / 10°	2 nm / 10°
**Polarization dependence**	Only LCP or RCP	Independent
**Fabrication requirements**	$\Delta\lambda$ = 1.6 nm resonance shift per nm unit cell size change at λ = 1500 nm	$\Delta\lambda$ = 1.3 nm resonance shift per nm unit cell size change at λ = 1280 nm
**Potential for active spatial light modulation**	Difficult	Straightforward

Supplementary Table 3 | Comparison to the state of the art of high-Q two dimensional wavefront manipulation. Comparison of the current state of the art of two-dimensional wavefront shaping with high quality factor considering key performance metrics for metasurface optical elements.

Quality factor: In non-local metasurfaces with uniform phase (i.e. no wavefront manipulation) the quality factor can be tuned to very large number following the relation $Q \sim 1/\delta$, where δ represents a

geometrical perturbation of the unicell[12]. However, when imprinting a phase profile on the metasurface the quality factor is largely reduced due to the bandstructure dispersion[12]. For this reason the quality factors that have been demonstrated for wavefront manipulation are limited, i.e. for 1D beam deflection/focusing up to $Q \leq 300$, and for 2D wavefront manipulation $Q \leq 86$ for a radial lens have been demonstrated[14]. In higher-order Mie-resonant metasurfaces, the quality factor is limited by the specific Mie-modes employed, here the ED/EO shows $Q \leq 668$. With other higher-order modes an increased Q may be attained. Furthermore, the high Q-factor is also preserved for wavefront manipulation. In practice, even higher wavefront-shaping quality factors are attained for beam deflection or focusing, as these depended on the specific alignment of the phase different resonators.

Numerical aperture: In non-local metasurfaces, the maximum attainable numerical aperture of an optical element is limited by the dispersion of the resonance wavelength by[12,13]

$$NA^2 < \frac{\omega_0}{k_0^2 |b| Q}. \tag{6}$$

For current designs, this limits the numerical aperture to < 0.26 with a quality factor of 86[13]. For higher quality factors the attainable numerical aperture further decreases. Higher-order Mie-resonant metasurfaces do not show a limit on numerical aperture for wavefront shaping, enabling the present demonstration of high-Q metalenses with numerical apertures of up to 0.8 and higher.

Limitation of efficiency: The overall efficiency of wavefront shaping with non-local metasurfaces is limited to < 25% due to their use of geometric phase and the resulting polarization conversion[12]. Higher efficiencies can be attained with multilayer metasurfaces, but these are difficult to realize experimentally at optical wavelengths, due to demanding overlay fabrication accuracies[15]. In higher-order Mie-resonant metasurfaces, there is no limit on the overall efficiency, and high efficiencies can be attained with simple single layer designs.

Incident angle dependence / dispersion: The bandstructure of non-local metasurfaces shows a dispersion that depends on the orientation of the incidence angle with respect to the perturbation in the structure. Along the direction of the perturbation (TM) the dispersion amounts to a resonance wavelength shift of ~70 nm/10° change in incidence angle. Along the orthogonal direction (TE), the dispersion amounts to a resonance wavelength shift of 9 nm/10° change in incidence angle[13]. Higher-

order Mie-resonant metasurfaces show a dispersion with resonance wavelength shift of less than 2 nm/10° change in incidence angle in both directions (see Fig. 1d-e). This shows that higher-order Mie-resonant metasurfaces are much less sensitive to illumination conditions and can operate with focused/diverging and oblique illumination.

Polarization dependence: Non-local metasurfaces require illumination with circularly polarized light of the correct handedness. This requires additional polarization optics for interfacing these surfaces with a light source. Furthermore, to reject unconverted light, a second polarizer is required in the detection. Higher-order Mie-resonant metasurfaces show a polarization-independent response and hence do not require additional polarization optics for integration.

Fabrication requirements: The fabrication requirements of high quality factor optical elements are generally demanding. Small changes in geometrical parameters of the unit cell can spectrally shift the optical resonance and hence affect the optical device performance, by resulting in errors in the scattered electric field amplitude and/or phase. In non-local metasurfaces this resonance shift is reported to be 1.6 nm wavelength per nanometer change in unit cell dimensions[13]. In higher-order Mie resonant metasurfaces this shift is 1.3 nm wavelength per nanometer change of the unit cell dimensions (see Supplementary Fig. 20). To attain efficient wavefront shaping with high quality factors (>600), both approaches require sub-nanometer accuracy of the fabricated structures. The present work shows that for higher-order Mie-resonant metasurfaces these fabrication requirements can be met with standard clean room fabrication techniques.

Potential for active spatial light modulation: Non-local metasurfaces employ Pancharatnam-Berry phase (also known as geometric phase) for wavefront manipulation, whereas higher-order Mie-resonant metasurfaces use resonance phase. For metasurfaces relying on geometric phase, the phase profile is permanently imprinted on the surface based on the fabricated geometry and cannot be arbitrarily reconfigured with an external input (e.g. by applying electrical voltage). While simple reconfiguration such as on-off switching or deformation of the phase profile has been demonstrated with non-local metasurfaces[14], an arbitrary reconfiguration of the phase is difficult for non-local metasurfaces, since it would require the in-plane re-orientation of the fabricated unit cells. Resonance-phase based metasurfaces have been shown to allow for such an arbitrary reconfiguration of the phase

profile (e.g. continuous beam steering) at up to microsecond time scales[16–19]. For higher-order Mie-resonant metasurfaces such a realization is straightforward, for example by using the materials with a thermo-optic, or electro-optic effect to spectrally shift the resonance and perform active phase modulation.

Finally, we compare our work to the previous work on one dimensional beam deflection with high quality factors. Supplementary Table 4 illustrates a comparison of key performance metrics of the state of the art of one dimensional high-quality factor beam deflection[11,20,21].

	Method	Q	Diffraction efficiency	Polarization dependence	Maximum deflection angle	Wavefront manipulation	Angular dispersion
Ref. 11	q-BIC	~25	35%*	Yes	43° (46° in glass, simulation)	1D	Not reported
Ref. 20	GMR	2500	15%	Yes	42° in air	1D	Large
Ref. 21	GMR	~380	56.7%	Yes	32° in air	1D	Large
This work	Higher order Mie resonances	1458	55.9%	No	35.4° in air	2D	Small

Supplementary Table 4 | Comparison to the state of the art of high-Q one dimensional wavefront manipulation. Comparison of the current state of the art of one-dimensional beam deflection with high quality factor considering key performance metrics for metasurface optical elements. Experimentally reported values are compared due to the crucial importance of fabrication imperfections when realizing these devices. The quality factor here represents the quality factor of 1D light deflection. Quality factors for Ref. 11 and 21 were approximated from figures. *As opposed to the other values of the diffraction efficiency, the value of Ref. 11 is normalized to the total incident light rather than total transmitted light. Experimental transmission is not reported in Ref. 11.

Supplementary References

1. Alaee, R., Rockstuhl, C. & Fernandez-Corbaton, I. An electromagnetic multipole expansion beyond the long-wavelength approximation. *Opt. Commun.* **407**, 17–21 (2018).

2. Yu, Y. F., Zhu, A. Y., Paniagua-Domínguez, R., Fu, Y. H., Luk'yanchuk, B. & Kuznetsov, A. I. High-transmission dielectric metasurface with 2π phase control at visible wavelengths. *Laser*

Photonics Rev. **9**, 412–418 (2015).

3. Savinov, V., Fedotov, V. A. & Zheludev, N. I. Toroidal dipolar excitation and macroscopic electromagnetic properties of metamaterials. *Phys. Rev. B - Condens. Matter Mater. Phys.* **89**, (2014).

4. Jeong, J., Goldflam, M. D., Campione, S., Briscoe, J. L., Vabishchevich, P. P., Nogan, J., Sinclair, M. B., Luk, T. S. & Brener, I. High Quality Factor Toroidal Resonances in Dielectric Metasurfaces. *ACS Photonics* **7**, 1699–1707 (2020).

5. Koshelev, K., Kruk, S., Melik-Gaykazyan, E., Choi, J. H., Bogdanov, A., Park, H. G. & Kivshar, Y. Subwavelength dielectric resonators for nonlinear nanophotonics. *Science* **367**, 288–292 (2020).

6. Rybin, M. V., Koshelev, K. L., Sadrieva, Z. F., Samusev, K. B., Bogdanov, A. A., Limonov, M. F. & Kivshar, Y. S. High- Q Supercavity Modes in Subwavelength Dielectric Resonators. *Phys. Rev. Lett.* **119**, 1–5 (2017).

7. Campione, S., Liu, S., Basilio, L. I., Warne, L. K., Langston, W. L., Luk, T. S., Wendt, J. R., Reno, J. L., Keeler, G. A., Brener, I. & Sinclair, M. B. Broken Symmetry Dielectric Resonators for High Quality Factor Fano Metasurfaces. *ACS Photonics* **3**, 2362–2367 (2016).

8. Tittl, A., Leitis, A., Liu, M., Yesilkoy, F., Choi, D. Y., Neshev, D. N., Kivshar, Y. S. & Altug, H. Imaging-based molecular barcoding with pixelated dielectric metasurfaces. *Science* **360**, 1105–1109 (2018).

9. Yesilkoy, F., Arvelo, E. R., Jahani, Y., Liu, M., Tittl, A., Cevher, V., Kivshar, Y. & Altug, H. Ultrasensitive hyperspectral imaging and biodetection enabled by dielectric metasurfaces. *Nat. Photonics* **13**, 390–396 (2019).

10. Benea-Chelmus, I. C., Mason, S., Meretska, M. L., Elder, D. L., Kazakov, D., Shams-Ansari, A., Dalton, L. R. & Capasso, F. Gigahertz free-space electro-optic modulators based on Mie resonances. *Nat. Commun.* **13**, 1–9 (2022).

11. Zhou, Y., Guo, S., Overvig, A. C. & Alù, A. Multiresonant Nonlocal Metasurfaces. *Nano Lett.* **23**,

6768–6775 (2023).

12. Overvig, A. C., Malek, S. C. & Yu, N. Multifunctional Nonlocal Metasurfaces. *Phys. Rev. Lett.* **125**, 17402 (2020).

13. Malek, S. C., Overvig, A. C., Alù, A. & Yu, N. Multifunctional resonant wavefront-shaping meta-optics based on multilayer and multi-perturbation nonlocal metasurfaces. *Light Sci. Appl.* **11**, 246 (2022).

14. Malek, S. C., Overvig, A. C., Shrestha, S. & Yu, N. Active nonlocal metasurfaces. *Nanophotonics* **10**, 655–665 (2021).

15. Overvig, A. & Alù, A. Wavefront-selective Fano resonant metasurfaces. *Adv. Photonics* **3**, 1–11 (2021).

16. Huang, Y. W., Lee, H. W. H., Sokhoyan, R., Pala, R. A., Thyagarajan, K., Han, S., Tsai, D. P. & Atwater, H. A. Gate-Tunable Conducting Oxide Metasurfaces. *Nano Lett.* **16**, 5319–5325 (2016).

17. Park, J., Jeong, B. G., Kim, S. Il, Lee, D., Kim, J., Shin, C., Lee, C. B., Otsuka, T., Kyoung, J., Kim, S., Yang, K. Y., Park, Y. Y., Lee, J., Hwang, I., Jang, J., Song, S. H., Brongersma, M. L., Ha, K., Hwang, S. W., Choo, H. & Choi, B. L. All-solid-state spatial light modulator with independent phase and amplitude control for three-dimensional LiDAR applications. *Nat. Nanotechnol.* **16**, 69–76 (2021).

18. Shirmanesh, G. K., Sokhoyan, R., Wu, P. C. & Atwater, H. A. Electro-optically Tunable Multifunctional Metasurfaces. *ACS Nano* **14**, 6912–6920 (2020).

19. Li, S. Q., Xu, X., Veetil, R. M., Valuckas, V., Paniagua-Domínguez, R. & Kuznetsov, A. I. Phase-only transmissive spatial light modulator based on tunable dielectric metasurface. *Science* **364**, 1087–1090 (2019).

20. Lawrence, M., Barton, D. R., Dixon, J., Song, J. H., van de Groep, J., Brongersma, M. L. & Dionne, J. A. High quality factor phase gradient metasurfaces. *Nat. Nanotechnol.* **15**, 956–961 (2020).

21. Lin, L., Hu, J., Dagli, S., Dionne, J. A. & Lawrence, M. Universal Narrowband Wavefront Shaping with High Quality Factor Meta-Reflect-Arrays. *Nano Lett.* **23**, 1355–1362 (2023).
